# Glanzmann's Thrombastenia: The Role of Tranexamic Acid in Oral Surgery

**DOI:** 10.1155/2018/9370212

**Published:** 2018-09-05

**Authors:** Rocco Franco, Michele Miranda, Laura Di Renzo, Antonino De Lorenzo, Alberta Barlattani, Patrizio Bollero

**Affiliations:** ^1^Section of Clinical Nutrition and Nutrigenomic, Department of Biomedicine and Prevention, School of Applied Medical-Surgical Sciences, University of Rome Tor Vergata, Via Montpellier 1, 00133 Rome, Italy; ^2^Department of Systems Medicine, Medical School, University of Rome Tor Vergata, Rome, Italy; ^3^Department of Clinical Sciences and Translational Medicine, University of Rome Tor Vergata, Rome, Italy

## Abstract

Glanzmann's thrombastenia (GT) is the most frequent inherited condition. GT is a genetic autosomal recessive disease caused by the alteration of the genes ITGA2B and ITGB3, located on the chromosome 17. The incidence of GT is calculated in 1 on 1000000. The patients, during their life, show episodes of mucocutaneous bleeding, epistaxis, and gingival bleeding. Some subjects required continuous bleeding transfusion. The aim of this case report is to demonstrate that oral assumption of tranexamic acid is a gold standard to prevent excessive bleeding. The patient GM of 36 years old with GT type 1 needs dental extractions of the teeth 4.7 and 4.8 at the “Tor Vergata” University Hospital in Rome. The specialist suggests that 3 days before surgery, the patient must take 6 vials every day of tranexamic acid that is used in obstetrics and gynecology. The teeth were extracted and applied suture. The patient is observed and is recommended mouth rinse with tranexamic acid. No bleeding complications were observed.

## 1. Introduction

Platelets are an important component in the hemostasis process. The damage of endothelium releases some proteins (i.e., collagen and thromboplastin) that avoid the aggregation of the platelet. The surface of the platelet permits the aggregation of procoagulant factor that activates the coagulation pathway. The platelet acquired disorders are more frequent in the clinical practice and are the result from the induction of the use of medications. The inherited conditions are very rare. The most frequent inherited condition is the Glanzmann's thrombastenia (GT). GT is a genetic autosomal recessive disease caused by the alteration of the genes ITGA2B and ITGB3, located on the chromosome 17. The gene codes for a platelet surface receptor GPIIb/GPIIIa, now known as ITG *α*IIb*β*3, that is, a large heterodimeric cell transmembrane receptor, comprised of a larger *α*IIb and a smaller *β*3 subunits. It is expressed in a large quantity on the surface of the platelet, up to 100000 copies. When a platelet is active, the integrin changes conformation and are exposed areas that bind fibrinogen and other soluble adhesive proteins such as Von Willenbrand factor. These proteins mediate the aggregation between a platelet in a Ca++ dependent manner [1]. The receptor also transmits signals into the platelet, mediates interactions with the cytoskeleton, and assures the transport of fibrinogen to alfa granules. The incidence of GT is calculated in 1/1000000. It is more common in some ethnic groups that have a high incidence of consanguinity such as Iraqui Jews and French Gypsies. Purpura is the initial manifestation of GT. The patients, during their life, showed episodes of mucocutaneous bleeding, epistaxis, and gingival bleeding. The subject, during invasive surgical acts or after accidental injuring, required bleeding transfusion. The gravity of bleeding is linked to the type of mutation. GT is classified into three subclasses (type 1, type 2, and other variant) characterized by the ability of platelets to retract a fibrin clot and store fibrinogen. Patients with type I have platelets with no ability to retract a fibrin clot and lack internal stores of fibrinogen; patients with type II have platelets with a reduced ability to retract a fibrin clot and low levels of platelet fibrinogen, while for other variant, the platelets have a differential ability to retract a fibrin clot and contain appreciable levels of platelet fibrinogen. The different types of GT have different quantities of integrin receptor: type I has <5% GPIIb±IIIa, type II 10 ± 25%, and other variant can have 100% of the normal receptor. Patients with GT need not take therapy daily but will always require treatment during surgical procedures, controlling bleeding after injury, and during spontaneous bleeding episodes. In general, the bleeding tendency in GT decreases with age. Local bleeding can be treated by local measures, such as fibrin sealants [2]. Regular dental care is essential to prevent gingival bleeding. Bleeding following trauma or surgical procedures could be severe, and transfusions are often given by precaution. For teeth extractions or for hemorrhage accompanying the loss of deciduous teeth, hemostasis can be significantly improved by the application of individually prepared plastic splints that provide physical support for hemostasis [3]. The correct management during dental surgery is very complex. The effectivity of transfusion is very discussed for negative effects. The administration of antifibrinolytic agents blocks the formation of fibrin and is a high-grade alternative [4]. Oral tranexamic acid is a method used in obstetrics and gynecology to treat menorrhagia. Oral surgery usually makes use of intravenous infusion or mouth rinse. The importance of the tranexamic acid use is discussed in a multitude of clinical studies. Tranexamic acid is an antifibrinolytic agent and inhibits the degradation of thrombus and consequently blocks bleeding. In dentistry, only the utilization of tranexamic acid mouth rinse is based on a significant literature [5]. Moreover, tranexamic acid does not require a period of hospitalization and consequently a great economic saving. The oral assumption is a new method utilized in oral surgery; only in obstetrics and gynecology, its assumption is based on important scientific literature. The aim of this case report is to describe the safety and practicality of oral tranexamic acid in dentistry and the total absence of adverse effects with respect to platelet concentrate.

## 2. Case Presentation

**Figure 1:**
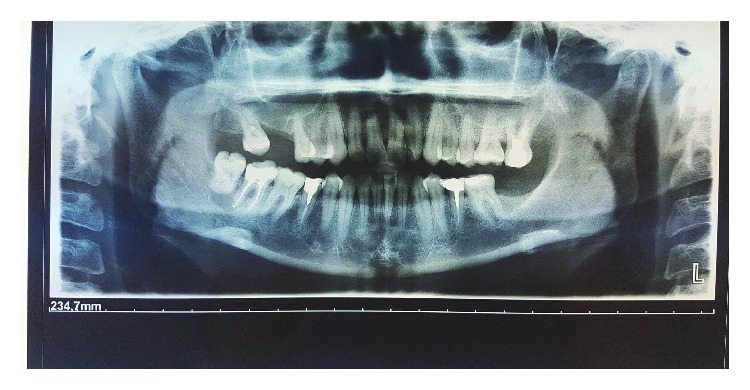
Initial radiography.

The patient GM of 36 years old with GT type 1 needs extractions of the dental teeth 4.7 and 4.8 for an endo-perio lesion, diagnosed by ortopanthomography, at the “Tor Vergata” University Hospital in Rome (Figure 1). Past laboratory tests revealed normal platelet counts and morphology, prolonged bleeding times, clotting time of 4.15 minutes, decreased clot retraction (20%), and abnormal platelet aggregation responses to physiologic stimuli such as adenosine diphosphate and epinephrine used during the aggregometer study. They have no family history of any bleeding disorder. The diagnosis occurred prematurely for the presence of frequent epistaxis, and the diagnosis was ascertained by light transmission aggregometry (LTA). The LTA evaluates shape change, lag phase, percent of aggregation, slope of aggregation, and deaggregation before and after the addition of an agonist (ADP, collagen, epinephrine, arachidonic acid, ristocetin, thrombin receptor-activating peptide, and thromboxane A2 mimetic). There was no history of consanguineous marriages in the family. The patient is looked after at the hematological center of Rome “Tor Vergata”. Preoperative examination was conducted by anesthesiologists. A hematological consult was requested. Oral surgery is classified as simple surgery because risk of bleeding is low. The hematology has advised against performing blood transfusions. The specialist suggests that 3 days before surgery, the patient must take by mouth 6 vials every day of tranexamic acid because the half-life of this drug is 2 hours [6]. The patient shows loss of multiple teeth and had executed other dental extraction with the same methods with no adverse events. A prophylactic assumption of amoxicillina and clavulanic acid has been started one day at the dose of 2 g daily before the surgery and continued for another 4 days.

**Figure 2:**
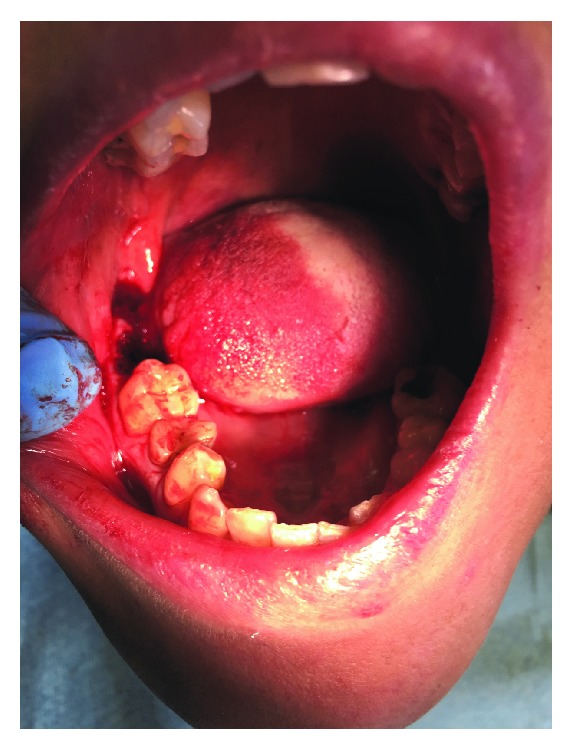
Postsurgery.

A peripheral block of the inferior alveolar nerve with bupivacaine without adrenaline and paraperiosteal anesthesia with articain and adrenaline in concentration 1/100000 was also performed. The surgery was conducted with atraumatic technique without osteotomies and mucoperiosteal flaps. The syndesmotomy of the elements 4.7 and 4.8 was performed; they were dislocated through a straight lever and extracted through an appropriate clamp (Figure 2). The socket was courted, washed with saline solution. At the end, oxidized regenerated cellulose was inserted in the socket, and the mucous membrane was sutured with silk 3-0.

Postoperative indications of oral surgery were given to the patient; she was monitored for about 4 hours postintervention. During the postoperatory time, an intravenous paracetamol infusion was performed because it does not interfere with the platelet aggregation. Mouth rinse with tranexamic acid was recommended to the patient. During the hospitalization, no major episode of bleeding was observed. The patient did not present any problems of any kind, and after 7 days, the sutures were removed. A maximum dose of 4 g daily of paracetamol is prescribed. Other nonsteroidal anti-inflammatory drugs are dissuaded because they stop the platelet aggregation. The patient did not present excessive and uncontrolled bleeding during the postoperative period and during the days following the operation.

## 3. Discussion

GT is a rare pathology that complicates surgical treatment for possible uncontrolled bleeding. A correct management of oral surgery in literature is the employment of endovenous tranexamic acid or platelet transfusion. Tranexamic acid, as shown in literature, works by slowing the breakdown of blood clots, which helps to prevent prolonged bleeding. It belongs to antifibrinolytic drugs. This drug is used to prevent excessive bleeding during menstrual period. Tranexamic acid is well tolerated; nausea and diarrhea are the most common adverse events. Increased risk of thrombosis with the drug has not been demonstrated in all clinical trials.

The blood or platelet transfusion in a GT patient has a high risk of alloimmunization (cross-reaction) against platelet glycoprotein IIb and/or IIIa. Specifically, the patient produce auto-antibodies against the glycoprotein. The occurrence of such alloantibodies is usually due to repeated blood transfusion and greatly complicates the treatment of these patients since they prevent effective platelet transfusion and might, theoretically, cause posttransfusion purpura [7].

Other rare reaction against transfusion observed in all patients are acute hemolytic reaction, allergic reaction, anaphylactic reaction, coagulation problems in massive transfusion, febrile nonhemolytic reaction, metabolic derangements, mistransfusion (transfusion of the incorrect product to the incorrect recipient), septic or bacterial contamination, transfusion-associated circulatory overload, transfusion-related acute lung injury, urticarial reaction, delayed hemolytic reaction, posttransfusion purpura, transfusion-associated graft-versus-host disease, transfusion-related immunomodulation, and infectious complications [8]. These complications are very rare, and the medical guidelines have greatly reduced these complications. In literature, only 16 studies treat the correct management during oral surgery of GT. Eight studies recommended platelet transfusion before oral surgery together with the use of endovenous tranexamic acid [9–16], four studies, in alternative to transfusion, recommended the use of recombinant-activated factor VII [17–20], one study recommended the use of plasma rich in platelet [21], and another the use of acrylic splint [22]. Two studies recommended the intravenous infusion of an antifibrinolytic agent [9, 23].

Oral surgery is classified as surgery with low risk of bleeding and are influenced by local factors such as inflammation and the grade of complexity. In this case report, we use the oral assumption of tranexamic acid for the presence of low adverse effects (nausea and diarrhea) compared to transfusion and practicality of employment. The patient had no adverse events and no excessive bleeding. The patient did not need blood transfusion. No cases in literature describe a similar method. This new method is more comfortable because the patient starts therapy domiciliary, and the oral surgery must carry out without the use of the platelet transfusion. The platelet transfusion required a period of hospitalization to observe possible adverse effects, and this is not an ambulatory procedure. Also, the transfusion has a higher economic cost. For this reason, the procedure with tranexamic acid can be performed in ambulatory. Only in obstetrics and gynecology, during menorrhagia, its assumption is based on important scientific literature. In gynecology, the recommended dose is 2–4.5 g/day. This drug is not influenced by food and has a half-life of 2 hours [24]. It is used also in orthopedic surgery [25]. The oral assumption of tranexamic acid shows a gold standard to prevent bleeding in patients affected by GT. New future studies must confirm our results with a major number of patients.
